# FlexiTerm: a flexible term recognition method

**DOI:** 10.1186/2041-1480-4-27

**Published:** 2013-10-10

**Authors:** Irena Spasić, Mark Greenwood, Alun Preece, Nick Francis, Glyn Elwyn

**Affiliations:** 1 School of Computer Science & Informatics, Cardiff University, Queen's Buildings, 5 The Parade, Cardiff, UK; 2 The Cochrane Institute for Primary Care and Public Health, Cardiff University, Heath Park, Cardiff, UK; 3 Dartmouth Center for Health Care Delivery Science, Dartmouth College, Hanover, NH, USA

## Abstract

**Background:**

The increasing amount of textual information in biomedicine requires effective term recognition methods to identify textual representations of domain-specific concepts as the first step toward automating its semantic interpretation. The dictionary look-up approaches may not always be suitable for dynamic domains such as biomedicine or the newly emerging types of media such as patient blogs, the main obstacles being the use of non-standardised terminology and high degree of term variation.

**Results:**

In this paper, we describe FlexiTerm, a method for automatic term recognition from a domain-specific corpus, and evaluate its performance against five manually annotated corpora. FlexiTerm performs term recognition in two steps: linguistic filtering is used to select term candidates followed by calculation of termhood, a frequency-based measure used as evidence to qualify a candidate as a term. In order to improve the quality of termhood calculation, which may be affected by the term variation phenomena, FlexiTerm uses a range of methods to neutralise the main sources of variation in biomedical terms. It manages syntactic variation by processing candidates using a bag-of-words approach. Orthographic and morphological variations are dealt with using stemming in combination with lexical and phonetic similarity measures. The method was evaluated on five biomedical corpora. The highest values for precision (94.56%), recall (71.31%) and F-measure (81.31%) were achieved on a corpus of clinical notes.

**Conclusions:**

FlexiTerm is an open-source software tool for automatic term recognition. It incorporates a simple term variant normalisation method. The method proved to be more robust than the baseline against less formally structured texts, such as those found in patient blogs or medical notes. The software can be downloaded freely at http://www.cs.cf.ac.uk/flexiterm.

## Background

Terms are means of conveying scientific and technical information [1]. More precisely, terms are linguistic representations of domain-specific concepts [2]. For practical purposes, they are often defined as phrases (typically nominal [3,4]) that frequently occur in texts restricted to a specific domain and have special meaning in a given domain. Terms are distinguished from other salient phrases by the measures of their unithood and termhood [4]. Unithood is defined as the degree of collocational stability (each term has a stable inner structure), while termhood refers to the degree of correspondence to domain-specific concepts (each term corresponds to at least one domain-specific concept). Termhood implies that terms carry heavier information load compared to other phrases used in a sublanguage, and as such they can be used to: provide support for natural language understanding, correctly index domain-specific documents, identify text phrases to be useful for automatic summarisation of domain-specific documents, efficiently skim through documents obtained through information retrieval, identify slot fillers for the information extraction tasks, etc. It is, thus, essential to build and maintain terminologies in order to enhance the performance of many natural language processing (NLP) applications.

### Automatic term recognition

Bearing in mind the potentially unlimited number of different domains and the dynamic nature of some domains (many of which expand rapidly together with the corresponding terminologies [5,6]), the need for efficient term recognition becomes apparent. Manual term recognition approaches are time-consuming, labour-intensive and prone to error due to subjective judgement. Therefore, automatic term recognition (ATR) methods are needed to efficiently annotate electronic documents with a set of terms they mention [7]. Note that here ATR refers to automatic extraction of terms from a domain-specific corpus [2] rather than matching a corpus against a dictionary of terms (e.g. [8]). Dictionary-based approaches are too static for dynamic domains such as biology or the newly emerging types of media such as blogs, where lay users may discuss topics from a specialised domain (e.g. medicine), but may not necessarily use a standardised terminology. Therefore, many biomedical terms cannot be identified in text using a dictionary look-up approach [9]. It is also important to differentiate between two related problems: ATR and keyphrase extraction. Both approaches aim to extract terms from text. The ultimate goal of ATR is to extract all terms from a corpus of documents, whereas keyphrase extraction targets only those terms that can summarise and characterise a single document. The two tasks will have similar approaches to candidate selection (e.g. noun phrases), after which the respective methods will diverge. Keyphrase extraction typically relies on supervised machine learning [10,11], while ATR is more likely to use unsupervised methods in order to explore the terminology space.

Manual term recognition is performed by relying on the conceptual knowledge, where human experts use tacit knowledge to identify terms by relating them to the corresponding concepts. On the other hand, ATR approaches resort to other types of knowledge that can provide clues about the terminological status of a given natural language utterance [12], e.g. morphological, syntactic, semantic and/or statistical knowledge about terms and/or their constituents (nested terms, words, morphemes). In general, there are two basic approaches to ATR [3]: linguistic (or symbolic) and statistical.

Linguistic approaches to ATR rely on the recognition of term formation patterns, but patterns alone are not sufficient for discriminating between terms and non-terms, i.e. there is no lexico-syntactic pattern according to which it could be inferred whether a phrase matching it is a term or not [2]. However, they provide useful clues that can be used to identify term candidates if not terms themselves. Linguistic ATR approaches usually involve pattern–matching algorithms to recognise candidate terms by checking if their internal syntactic structure conforms to a predefined set of morpho-syntactic rules [13], e.g. *cyclic/JJ adenosine/NN monophosphate/NN* matches the pattern (*JJ | NN*)^+^*NN* (*JJ* and *NN* are part-of-speech tags used to denote adjectives and nouns respectively). Others simply focus on noun phrases of certain length: 2 (word bigrams), 3 (word trigrams) and 4 (word quadgrams) [14]. However, both approaches depend strongly on the ability to reliably identify noun phrases, a task that has proven to be problematic in the biological domain mainly due to the lack of highly accurate part-of-speech (POS) taggers for biomedical text [15].

Statistical ATR methods rely on the following hypotheses regarding the usage of terms [4]: specificity (terms are likely to be confined to a single or few domains), absolute frequency (terms tend to appear frequently in their domain), and relative frequency (terms tend to appear more frequently in their domain than in general). In most of the methods, two types of frequencies are used: frequency of occurrence in isolation and frequency of co-occurrence. One of the measures that combines this information is mutual information, which can be used to measure the unithood of a candidate term, i.e. how strongly its constituents are associated with one another [16]. Similarly, the Tanimoto's coefficient can be used to locate the words that appear more frequently in co-occurrence than isolated [17]. Statistical approaches are prone to extracting not only terms, but also other types of collocations: functional, semantic, thematic and other [18]. This problem is typically remedied by employing linguistic filters in the form of morpho-syntactic patterns in order to extract candidate terms from a corpus, which are then ranked using statistical information. A popular example of such an approach is C-value [19], a method which combines linguistic knowledge and statistical analysis. First, POS tagging is performed, since the syntactic information is needed in order to apply syntactic pattern matching against a corpus. The role of these patterns is to extract only those words sequences that conform to syntactic rules that describe a typical inner structure of terms. In the statistical part of the C-value method, each term candidate is quantified by its termhood following the idea of a cost-criteria based measure originally introduced for automatic collocation extraction [20]. C-value is calculated as a combination of the term’s numerical characteristics: length as the number of tokens, absolute frequency and two types of frequencies relative to the set of candidate terms containing the nested candidate term (frequency of occurrence nested inside other candidate terms and the number of different term candidates containing the nested candidate term). Formally, if *T* is a set of all candidate terms, *t* ∈ *T*, | *t* | is the number of words in *t*, *f: T* → *N* is the frequency function, *P*(*T*) is the power set of *T*, *S: T* → *P*(*T*) is a function that maps a candidate term to the set of all other candidate terms containing it as a substring, then the termhood, denoted as *C-value*(*t*), is calculated as follows:

(1) $$C-\mathrm{value}\left(t\right)=\left\{{}_{\mathrm{In}\;\left|t\right|\cdot (f\left(t\right)-\frac{1}{\left|S\left(t\right)\right|}\sum_{s\in S\left(t\right)}f\left(s\right))}^{\mathrm{In}\;\left|t\right|\cdot f\left(t\right)}{\;}_{,\mathrm{if}\;S\;\left(t\right)\neq \emptyset }^{,\mathrm{if}\;S\;\left(t\right)=\emptyset }\right)$$

The method favours longer, more frequently and independently occurring term candidates. Better results have been reported when the limited paradigmatic modifiability was used as a measure of termhood, which is based on the probability with which specific slots in a term candidate can be filled by other tokens, i.e. the tendency not to let other tokens occur in particular slots [14].

### Term variation

Both methods will perform well to identify terms that are used consistently in the corpus, i.e. where their occurrences do not vary in structure and content. However, terms typically vary in several ways:

– morphological variation, where the transformation of the content words involves inflection (e.g. *lateral meniscus* vs. *lateral menisci*) or derivation (e.g. *meniscal tear* vs. *meniscus tear*),

– syntactic variation, where the content words are preserved in their original form (e.g. *stone in kidney* vs. *kidney stone*),

– semantic variation, where the transformation of the content words involves a semantic relation (e.g. *dietary supplement* vs. *nutritional supplement*).

It is estimated that approximately one third of an English scientific corpus accounts for term variants, the majority of which (approximately 59%) are semantic variants, while morphological and syntactic variants account for around 17% and 24% respectively [1]. The large number of term variants emphasises the necessity for ATR to address the problem of term variation. In particular, statistically based ATR methods should include term normalisation (the process of associating term variants with one another) in order to aggregate occurrence frequencies at the semantic level rather than dispersing them across separate variants at the linguistic level [21].

Lexical programs distributed with the UMLS knowledge sources [22] incorporate an effective method for neutralising term variation [23]. Orthographic, morphological and syntactic term variants are normalised simply by tokenising each term, lowercasing each token, converting each word to its base form (lemmatisation), ignoring punctuation, ignoring tokens shorter than three characters, removing stop words (i.e. common English words such as *of, and, with* etc.) and sorting the remaining tokens alphabetically. For example, the genitive (possessive) forms are neutralised by this approach: *Alzheimer’s disease* is first tokenised to (*Alzheimer,’ , s, disease*), then lowercased (*alzheimer,’ , s, disease*), after which punctuation and short tokens are removed, and the remaining tokens finally sorted to obtain the normalised term representative (*alzheimer, disease*). The normalisation of the variant *Alzheimer disease* results in the same normalised form, so the two variants are matched through their normalised forms. Similarly, the genitive usage of the preposition *of* can be neutralised. For example, *aneurysm of splenic artery* and *splenic artery aneurysm* share the same normalised form. Note that such an approach may lead to overgeneralisation, e.g. *Venetian blind* and *blind Venetian* vary only in order, but have unrelated meanings. However, few such examples have been reported in practice [23]. Derivational and inflectional variation of individual tokens is addressed by rules which define mapping between suffixes across different lexical categories. For example, the rule *–a|NN|–al|JJ* maps between nouns ending with *–a* and adjectives ending with *–al* that match on the remaining parts (e.g. *bacteria* and *bacterial*), while the rule *–us|NN|–i|NN* matches inflected noun forms that end with *–us* and *–i* (e.g. *fungus* and *fungi*).

## Methods

### Method overview

FlexiTerm is an open-source, stand-alone application developed to address the task of automatically identifying terms in textual documents. Similarly to C-value [24], our approach performs term recognition in two stages. First, lexico–syntactic information is used to select term candidates, after which term candidates are scored using a formula that estimates their collocation stability, but taking into account possible syntactic, morphological, derivational and orthographic variation. What differentiates FlexiTerm from C-value is the flexibility with which term candidates are compared to one another. Namely, C-value relies on exact token matching to measure the overlap between term candidates in order to identify the longest collocationally stable phrases, also taking into account the exact order in which these tokens occur. The order condition has been relaxed in later versions of C-value in order to address the term variation problem using transformation rules to explicitly map between different types of syntactic variants (e.g. *stone in kidney* is mapped to *kidney stone* using the rule *NN*_*1*_*PREP NN*_*2*_ *→ NN*_*2*_*NN*_*1*_) [25]. FlexiTerm uses flexible comparison of term candidates by treating them as bags of words, thus completely ignoring the order of tokens, following a more pragmatic approach to neutralising term variation, which has been successfully used in practice [23] (see the Background section for details). Still, the C-value approach relies on exact token matching, which may be too rigid for types of documents that are prone to typographical errors and spelling mistakes, e.g. medical notes [26] and patient blogs [27]. Therefore, FlexiTerm adds additional flexibility to term candidate comparison by allowing approximate token matching based on lexical and phonetic similarity, which often indicates not only semantically equivalent words (e.g. *hemoglobin* vs. *haemoglobin*), but also semantically related ones (e.g. *hypoglycemia* vs. *hyperglycemia*).

Edit distance (ED) has been widely applied in NLP for approximate string matching, where the distance between identical strings is equal to zero and it increases as the strings get more dissimilar with respect to the characters they contain and the order in which they appear. ED is defined as the minimal number (or cost) of changes needed to transform one string into the other. These changes may include the following edit operations: insertion of a single character, deletion of a single character, replacement (substitution) of two corresponding characters in the two strings being compared, and transposition (reversal or swap) of two adjacent characters in one of the strings [28]. This approach has been successfully utilised in NLP applications to deal with alternate spellings, misspellings, the use of white spaces as means of formatting, the use of upper- and lower-case letters and other orthographic variations. For example, 80% of the spelling mistakes can be identified and corrected automatically by considering a single omission, insertion, substitution or reversal [28]. ED can be practically computed using a dynamic programming approach [29]. FlexiTerm applies ED to improve token matching, thus allowing different morphological, derivational and orthographic variants together with statistical information attached to them to be aggregated.

### Linguistic pre-processing

Our approach to ATR takes advantage of lexico–syntactic information to identify term candidates. Therefore, the input documents need to undergo linguistic pre–processing in order to annotate them with relevant lexico–syntactic information. This process includes sentence splitting, tokenisation and POS tagging. Practically, text is first processed using the Stanford log-linear POS tagger [30,31], which splits text into sentences and tokens, which are then annotated with POS information, i.e. lexical categories such as noun, verb, adjective, etc. The output of linguistic pre-processing is a document in which sentences and lexical categories of individual tokens (e.g. nouns, verbs, etc.) are marked up. We used the Penn Treebank tag set [32] throughout this article (e.g. *NN*, *JJ*, *NP*, etc.).

### Term candidate extraction and normalisation

Once input documents have been pre-processed, term candidates are extracted by matching patterns that specify the syntactic structure of targeted noun phrases (NPs). These patterns are the parameters of the method and may be modified if needed. In our experiments, we used the following three patterns:

1. (*JJ | NN*)^+^*NN*, e.g. *chronic obstructive pulmonary disease*

2. (*NN* | *JJ*)* *NN POS* (*NN* | *JJ*)* *NN*, e.g. *Hoffa's fat pad*

3. (*NN* | *JJ*)* *NN IN* (*NN* | *JJ*)* *NN*, e.g. *acute exacerbation of chronic bronchitis*

Further, lexical information is used to improve boundary detection of term candidates by trimming leading and trailing stop words, which include common English words (e.g. *any*), but also frequent modifiers of biomedical terms (e.g. *small* in *small Baker's cyst*).

In order to neutralise morphological and syntactic variation, all term candidates are normalised. The normalisation process is similar to the one described in [23] and consists of the following steps: (1) Remove punctuation (e.g. ' in possessives), numbers and stop words including prepositions (e.g. *of*) (2) Remove any lowercase tokens with ≤2 characters. (3) Stem each remaining token. For example, this process would map term candidates such as *hypoxia at rest* and *resting hypoxia* to the same normalised form {*hypoxia, rest*}, thus neutralising both morphological and syntactic variation resulting in two linguistic representations of the same medical concept. The normalised candidate is used to aggregate the relevant information associated with the original candidates, e.g. their frequency of occurrence. This means that subsequent calculation of termhood is performed against normalised term candidates.

It should be noted that the step 2 removes only lowercase tokens. This approach effectively removes possessive *s* in *Baker's cyst*, but not *D* in *vitamin D* as uppercase tokens generally convey more important information, which is therefore preserved in this approach. Also note that removing tokens longer than 2 characters would be too aggressive in deleting not only possessives and some prepositions (e.g. *of*), but also essential term constituents as it would be the case with *fat pad*, in which both tokens would be lost, thus completely ignoring it as a potential term.

### Token-level similarity

While many types of morphological variation are effectively neutralised with stemming used as part of the normalisation process (e.g. *transplant* and *transplantation* will be reduced to the same stem), exact token matching will still fail to match synonyms that differ due to orthographic variation (e.g. *haemorrhage* and *hemorrhage* are stemmed to *haemorrhag* and *hemorrhag* respectively). On the other hand, such variations can be easily identified using approximate string matching. For example, the ED between the two stems is only 1 – a single insertion of the character *a*: *h[a]emorrhag*. In general, token similarity can be used to boost the termhood of related terms by aggregating statistical information attached to them. For example, when terms such as *asymptomatic HIV infection* and *symptomatic HIV infection* are considered separately, the frequency of nested term *HIV infection*, which also occurs independently, will be much greater than that of either of the longer terms. This introduces a strong bias towards shorter terms (often a hypernym of the longer terms), which may cause longer terms not to be identified as such, thus overgeneralising the semantic content. However, the lexical similarity between the constituent tokens *asymptomatic* and *symptomatic* (one deletion operation) combined with the other two identical tokens indicates high similarity between the candidate terms, which can be used to aggregate the associated information and reduce the bias towards shorter terms.

The normalisation process continues by expanding previously normalised term candidates with similar tokens found in the corpus. In the previous example, the two normalised candidates {*asymptomat, hiv, infect*} and {*symptomat, hiv, infect*} would both be expanded to the same normalised form {*asymptomat, symptomat, hiv, infect*}. In our implementation, similar tokens are identified based on their phonetic and lexical similarity calculated with Jazzy [33] (a spell checker API). Jazzy is based on ED [28] described earlier in more detail, but it also includes two more edit operations to swap adjacent characters and to change the case of a letter. Apart from string similarity, Jazzy supports phonetic matching with the Metaphone algorithm [34], which aims to match words that sound similar without necessarily being lexically similar. This capability is important in dealing with new phenomena such as SMS language, in which the original words are often replaced by phonetically similar ones to achieve brevity (e.g. *l8* and *late*). This phenomenon is becoming increasingly present in online media (e.g. patient blogs) and needs to be taken into account in modern NLP applications.

### Termhood calculation

The termhood calculation is based on the C-value formula given in (1) [19]. A major difference in relation to the original C-value method is the way in which term candidates are normalised. In the C-value approach the notion of nestedness, as part of determining the set *S*(*t*), is based on substrings nested in a term candidate *t* treated as a string. In our approach, a term candidate is treated as a bag of words, which allows nestedness to be determined using subsets instead of substrings. This effectively bypasses the problem of syntactic variation, where individual tokens do not need to appear in the same order (e.g. *kidney stone* vs. *stone in kidney*). Other causes of term variability (mainly morphological and orthographic variation) are addressed by automatically adding similar tokens to normalised term candidates, which means that nestedness can be detected between lexically similar phrases using the subset operation. For example, exact matching would fail to detect *posterolateral corner* as nested in *postero-lateral corner sprain* because of hyphenation (a special case of orthographic variation). In our approach, these two term candidates would be represented as {*postero-later, posterolater, corner*} and {*postero-later, posterolater, corner, sprain*} respectively, where similar stems *postero-later* and *posterolater* have been automatically detected in the corpus and used to expand normalised term candidates. In this case, nestedness is detected by simply checking the following condition: {*postero-later, posterolater, corner*} ⊆ {*postero-later, posterolater, corner, sprain*}.

The FlexiTerm method is summarised with the following pseudocode:

1. Pre-process text to annotate it with lexico-syntactic information.

2. Select term candidates using pattern matching on POS tagged text.

3. Normalise term candidates by performing the following steps.

a. Remove punctuation, numbers and stop words.

b. Remove any lowercase tokens with ≤2 characters.

c. Stem each remaining token.

4. Extract distinct token stems from normalised term candidates.

5. Compare token stems using lexical and phonetic similarity calculated with Jazzy API.

6. Expand normalised term candidates by adding similar token stems determined in step 5.

7. For each normalised term candidate *t*:

a. Determine set *S*(*t*) of all normalised term candidates that contain *t* as a subset.

b. Calculate *C-value*(*t*) according to formula (1).

8. Rank normalised term candidates using their C-value.

### Output

**Figure 1:**
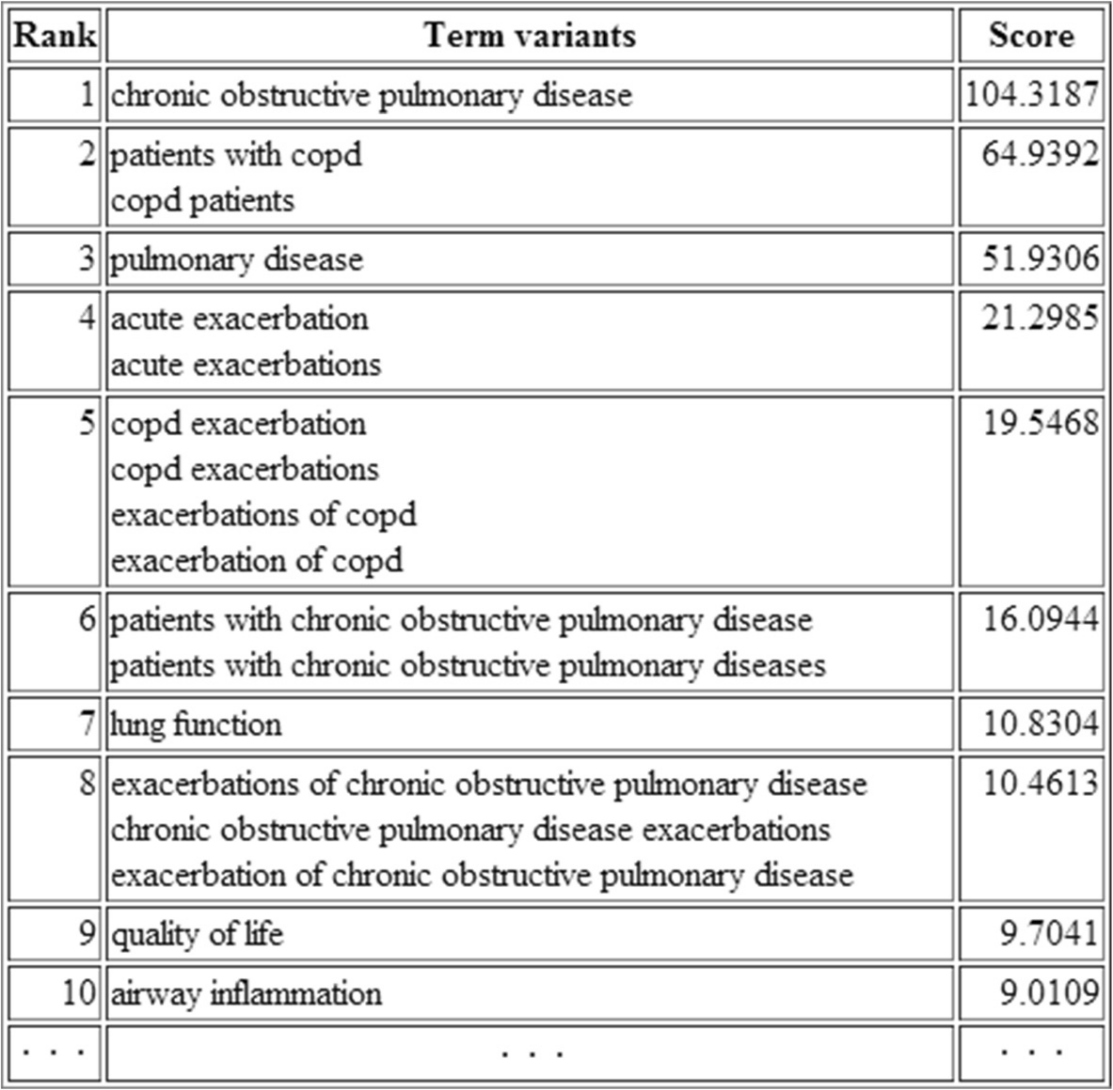
**Sample output of FlexiTerm.** A ranked list of terms and their variants based on their termhood scores.

**Figure 2:**
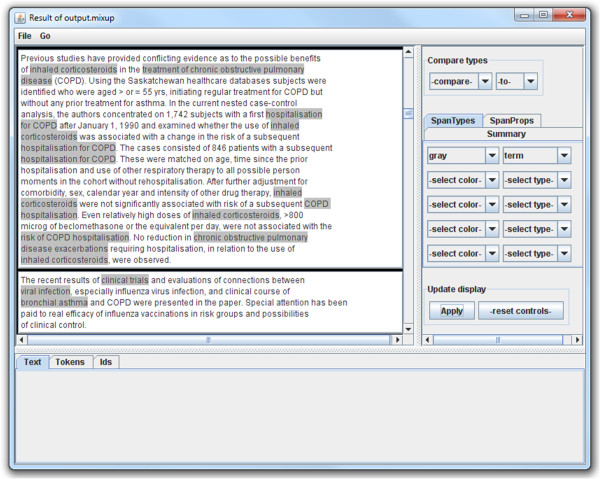
**Annotated occurrences of terms recognised by FlexiTerm.** The annotations are visualised using MinorThird.

Once terms are recognised, FlexiTerm produces output that can be used by either a human user or other NLP applications. Three types of output are produced: (1) a ranked list of terms with their termhood scores presented as table in the HTML format, (2) a plain list of terms that can be utilised as a lexicon by other NLP applications, and (3) a list of regular expressions in Mixup (My Information eXtraction and Understanding Package), a simple pattern-matching language [35]. Figure 1 shows a portion of the HTML output in which term variants with the same normalised form are grouped together and assigned a single termhood score. Lowercased term variants are given as they occurred in the corpus and are ordered by their frequency of occurrence. In effect, the plain text output presents the middle column of the HTML output. The term list can be utilised in a dictionary matching approach (e.g. [36]) to annotate all term occurrences in a corpus. Rather than annotating occurrences in text, we opted for this approach as it is more flexible and avoids conflict with other annotations produced by other applications. Still, for quick overview of terms and the context in which they appeared, the Mixup output can be used by MinorThird, a collection of Java classes for annotating text [35], to visualise the results (see Figure 2) and save the stand-off annotations, which include document name, start position of a term occurrence and its length.

## Results

### Data

FlexiTerm is a domain independent ATR method, that is – it does not rely on any domain specific knowledge (e.g. rules or dictionaries) to recognise terms in a domain specific corpus. A comprehensive study of subdomain variation in biomedical language has highlighted significant implications for NLP applications, in particular standard training and evaluation procedures for biomedical NLP tools [37]. This study revealed that the commonly used molecular biology subdomain is not representative of the overall biomedical domain, meaning that the results obtained using a corpus from this subdomain (e.g. [38]) cannot be generalised in terms of expecting comparable performance with other types of biomedical text. In particular, a comparative evaluation of ATR algorithms indicated that choice, design, quality and size of corpora have a significant impact on their performance [39]. Therefore, in order to demonstrate the portability of our method across sublanguages, i.e. languages confined to specialised domains [40], we used multiple data sets from different biomedical subdomains (e.g. molecular biology, medical diagnostic imaging or respiratory diseases) as well as text written by different types of authors and/or aimed at different audience (e.g. scientists, healthcare professionals or patients). We used five data sets (see Tables 1 and 2 for basic description).

**Table 1:** Data sets used in evaluation

**Data set**	**Topic**	**Document type**	**Source**	**Search terms**
1	molecular biology	abstract	PubMed	*human*, *blood cell*, *transcription factor*
2	COPD	abstract	PubMed	"*pulmonary disease, chronic obstructive*" [MeSH Terms]
3	COPD	blog post	Web	*COPD, chronic obstructive* {*pulmonary | lung | airways | respiratory*} *disease, bronchitis, emphysema*
4	obesity, diabetes	clinical narrative	i2b2	N/A
5	knee MRI scan	clinical narrative	NHS	N/A

Qualitative description of the corpora.

**Table 2:** Data sets used in evaluation

**Data set**	**Size (KB)**	**Documents**	**Sentences**	**Tokens**	**Distinct tokens**	**Distinct stems**
1	145	100	906	24,096	3,430	2,720
2	150	100	949	26,174	3,837	3,049
3	169	100	1,949	40,461	4,404	3,422
4	300	100	3,022	55,845	5,402	4,504
5	73	100	960	13,093	946	824

Quantitative description of the corpora.

Data set 1 refers to 100 documents randomly selected from GENIA, a semantically annotated corpus for NLP applications, which consists of molecular biology abstracts retrieved from the PubMed database using *human*, *blood cell* and *transcription factor* as search terms [38]. Similarly, data set 2 consists of 100 abstracts retrieved from PubMed, but on a different topic. Unlike data set 1, which belongs to biomolecular domain, data set 2 belongs to clinical domain, more specifically chronic obstructive pulmonary disease (COPD), and it has been collected using the following PubMed query: *"pulmonary disease, chronic obstructive" [MeSH Terms]*. This distinction is relevant given the considerable differences between biomolecular and clinical sublanguages [41].

Furthermore, apart from topical difference, we wanted to explore differences in the writing style. Therefore, we collected text data from the same clinical domain (i.e. COPD), but written by non-medical experts, i.e. patients or caregivers. Data set 3 represents a collection of 100 blog posts, which have been collected from blogs identified with blog search engines (Google Blog Search and Technorati) using a set of COPD–related search terms. Query results were reviewed manually in order to identify blogs with patient contributions and exclude blogs written by medical practitioners or those set up for marketing purposes.

Finally, we wanted to contrast the clinical sublanguage used in clinical practice against that used in scientific literature (see data set 2). Lexical analysis of a large corpus of various types of medical records (discharge summaries, radiology reports, progress notes, emergency room reports and letters) revealed that clinical narratives are characterised by a high degree of misspellings, abbreviations and idioms and as such pose considerable challenges for NLP applications [26]. A particular challenge for ATR, especially when dictionary-based, is the fact that over 20% of the words in the given corpus were unrecognisable i.e. were not recognizable medical words, common words or names, and could not be algorithmically or contextually converted to such words. Almost 78% of unrecognisable words were judged to be probably correctly spelled medical words. To test the flexibility of our method in coping with irregularities of clinical sublanguages, we used two additional data sets, which were anonymized prior to their distribution. Data set 4 represents a collection of narratives extracted from hospital discharge summaries of patients with history of obesity or diabetes, which were distributed for the i2b2 Challenge in NLP for Clinical Data [42]. Hospital discharge summaries were split into sections by matching the most frequent keywords used in section titles [43], after which the narrative sections referring to history of present illness and hospital course were extracted automatically. Finally, data set 5 represents a collection of magnetic resonance imaging (MRI) reports acquired from a National Health Service (NHS) hospital. They describe knee images taken following an acute injury.

### Gold standard

Terms, defined here as noun phrases referring to concepts relevant in a considered domain, were annotated by two independent annotators (labelled A and B in Tables 3, 4, 5, 6, 7, 8). The annotation exercise was performed using MinorThird, a collection of Java classes for annotating text [35]. Each annotated term was automatically tokenised in order to enable token-level evaluation later on (see the following subsection for details). Therefore, the annotation task resulted in each token being annotated as being part of a term, either single or multi word.

**Table 3:** Contingency tables for inter–annotator agreement

		**B**		
		**Yes**	**No**	**Total**
**A**	**Yes**	*n*_11_	*n*_12_	*n*_1._
	**No**	*n*_21_	*n*_22_	*n*_2._
	**Total**	*n*_.1_	*n*_.2_	*N*
		**B**		
		**Yes**	**No**	**Total**
**A**	**Yes**	*p*_11_	*p*_12_	*p*_1._
	**No**	*p*_21_	*p*_22_	*p*_2._
	**Total**	*p*_.1_	*p*_.2_	*p*

General structure of a contingency table, where *n* and *p* annotate the total numbers and proportions respectively.

**Table 4:** Contingency tables for inter–annotator agreement on data set 1

		**B**		
		**Yes**	**No**	**Total**
**A**	**Yes**	11,948	346	12,294
	**No**	1,664	10,138	11,802
	**Total**	13,612	10,484	24,096
		**B**		
		**Yes**	**No**	**Total**
**A**	**Yes**	0.496	0.014	0.510
	**No**	0.069	0.421	0.490
	**Total**	0.565	0.435	1

Agreement at the token level.

**Table 5:** Contingency tables for inter–annotator agreement on data set 2

		**B**		
		**Yes**	**No**	**Total**
**A**	**Yes**	7,256	1,100	8,356
	**No**	1,062	16,756	17,818
	**Total**	8,318	17,856	26,174
		**B**		
		**Yes**	**No**	**Total**
**A**	**Yes**	0.277	0.042	0.319
	**No**	0.041	0.640	0.681
	**Total**	0.318	0.682	1

Agreement at the token level.

**Table 6:** Contingency tables for inter–annotator agreement on data set 3

		**B**		
		**Yes**	**No**	**Total**
**A**	**Yes**	2,325	204	2,529
	**No**	436	37,496	37,932
	**Total**	2,761	37,700	40,461
		**B**		
		**Yes**	**No**	**Total**
**A**	**Yes**	0.057	0.005	0.062
	**No**	0.011	0.927	0.938
	**Total**	0.068	0.932	1

Agreement at the token level.

**Table 7:** Contingency tables for inter–annotator agreement on data set 4

		**B**		
		**Yes**	**No**	**Total**
**A**	**Yes**	14,396	1,454	15,850
	**No**	2,269	37,726	39,995
	**Total**	16,665	39,180	55,845
		**B**		
		**Yes**	**No**	**Total**
**A**	**Yes**	0.258	0.026	0.284
	**No**	0.040	0.676	0.716
	**Total**	0.298	0.702	1

Agreement at the token level.

**Table 8:** Contingency tables for inter–annotator agreement on data set 5

		**B**		
		**Yes**	**No**	**Total**
**A**	**Yes**	5,312	278	5,590
	**No**	252	7,251	7,503
	**Total**	5,564	7,529	13,093
		**B**		
		**Yes**	**No**	**Total**
**A**	**Yes**	0.406	0.021	0.427
	**No**	0.019	0.554	0.573
	**Total**	0.425	0.575	1

Agreement at the token level.

Cohen's Kappa coefficient [44] was used to measure the inter-annotator agreement. After producing contingency tables following the structure described in Table 3, the Kappa coefficient was calculated according to the following formula:

$$\kappa =\frac{A_{o}-A_{e}}{1-A_{e}}$$

where *A*_*o*_ = *p*_11_ + *p*_22_ is observed agreement and *A*_*e*_ = *p*_1._·*p*_.1_ + *p*_2._·*p*_.2_ is expected agreement by chance. The Kappa coefficient of 1 indicates perfect agreement, whereas 0 indicates chance agreement. Therefore, higher values indicate better agreement. Different scales have been proposed to interpret the Kappa coefficient [45,46]. In most interpretations, the values over 0.8 are generally agreed to indicate almost perfect agreement.

Based on the contingency tables produced for each data set (see Tables 4, 5, 6, 7, 8), we calculated the Kappa coefficient values given in Table 9, which ranged from 0.809 to 0.918, thus indicating very high agreement. Gold standard for each data set was then created as the intersection of positive annotations. In other words, gold standard represents a set of all tokens that were annotated as being part of a domain-specific term by both annotators.

**Table 9:** Inter–annotator agreement

**Data set**	**Observed agreement (*A***_***o***_**)**	**Expected agreement (*A***_***e***_**)**	**Kappa coefficient (*κ *)**
1	0.917	0.501	0.834
2	0.917	0.566	0.809
3	0.984	0.878	0.869
4	0.934	0.587	0.840
5	0.960	0.511	0.918

The values of three agreement measures.

**Figure 3:**
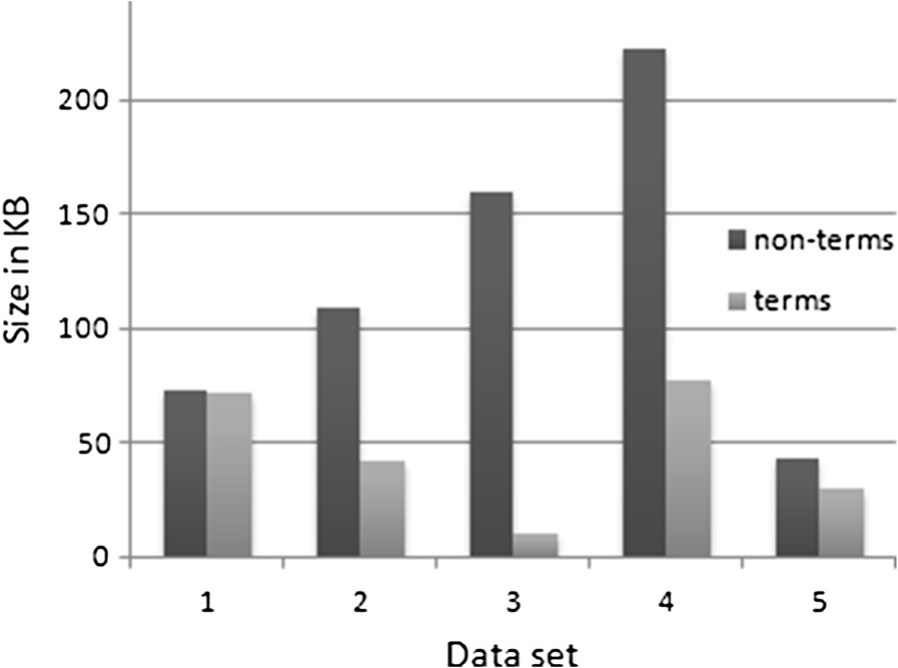
**The size and distribution of data sets.** Comparison of terminological and non terminological content.

The extent of terminological content across the five data sets illustrates great variation in biomedical language and justifies the need for multiple data sets in order to generalise the results [37]. To illustrate this point we converted the information from Tables 4, 5, 6, 7, 8 to a histogram shown in Figure 3. Terms account for a massive 50% in PubMed abstracts in molecular biology (data set 1), whereas the same type of documents in medicine (data set 2) includes 28% of terminological content. Not surprisingly, terms account for only 6% in medical information reported by laymen (data set 3). Finally, the terminological content of medical notes also varies significantly with 26% in hospital discharge summaries (data set 4) compared to 41% in radiology reports (data set 5). These variations should be kept in mind later on when the evaluation results for the top *k* automatically recognised terms are reported (*k* = 10, 20, …, 500).

### Evaluation measures

ATR can be viewed as an information extraction (IE) task, where term occurrences constitute information to be extracted from text, and thus can be evaluated using the contingency table model [47]. Information extracted by the system is classified either as a true positive if it is indeed a term or as a false positive if it is not. Conversely, each term occurrence is classified as a false negative if it is not extracted by the system. Given the total numbers of true positives (*TP*), false positives (*FP*) and false negatives (*FN*), precision (*P*) and recall (*R*) are calculated as the following ratios:

$$P=\frac{\mathrm{TP}}{\mathrm{TP}+\mathrm{FP}}R=\frac{\mathrm{TP}}{\mathrm{TP}+\mathrm{FN}}$$

In other words, precision represents the proportion of correctly extracted term occurrences, while recall represents the proportion of term occurrences that are extracted by the system. Given the precision and recall values, F-measure is calculated as their harmonic mean:

$$F=\frac{2\cdot P\cdot R}{P+R}$$

An important question that remains to be answered is what counts as a correctly recognised term. It is natural to assume that it would match an annotated term occurrence exactly. Such an approach is suitable for common IE task such as named entity recognition (e.g. protein name recognition), where it is easier to define the exact boundaries of the names occurring in text. However, it is less suitable for ATR, since terms are often formed by combining other terms. Consider for example a term such as *protein kinase C activation pathway*, where *protein*, *protein kinase*, *protein kinase C*, *activation*, *pathway, protein activation pathway* and *protein kinase C activation pathway* are all terms defined in the UMLS [22]. This fact makes the annotation task more complex and consequently more subjective. Even if we simplified the task by focusing only on the most specific concepts, i.e. the ones described by the longest term encompassing all other nested terms, it would be difficult to justify the recognition of subsumed terms as term recognition errors.

For these reasons, it may be more appropriate to apply token-level evaluation, which effectively evaluates the degree of overlap between automatically extracted terms and those manually annotated in the gold standard. Similar approach has been used for IE evaluation in i2b2 NLP challenges [48], as it may provide more detailed insight into the IE performance. We adapted this approach for ATR evaluation to calculate token-level precision and recall. The same contingency table model is applied to individual tokens that are part of term occurrences either automatically extracted by the system or manually annotated in the gold standard. Each token extracted as part of a presumed term is classified as a true positive if it is annotated in the gold standard; otherwise it is classified as a false positive. Similarly, each token annotated in the gold standard is classified as a false negative if it is not extracted by the system as part of an automatically recognised term. Precision, recall and F-measure are then calculated as before.

### Evaluation results and discussion

**Figure 4:**
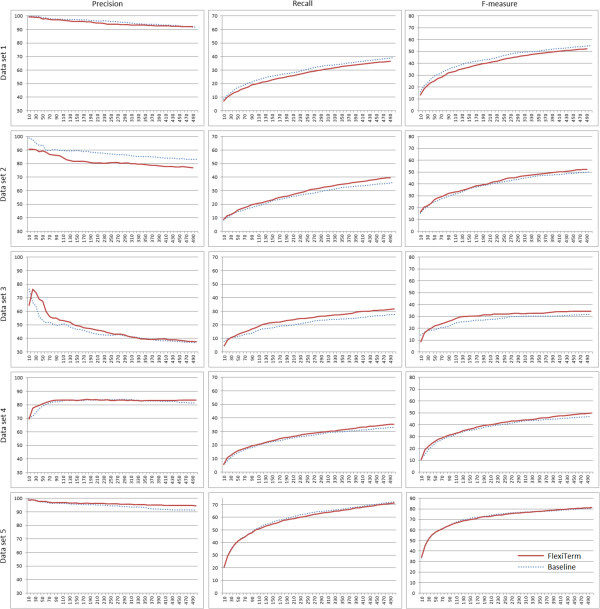
**Evaluation results.** Comparison to the baseline method with respect to the precision, recall and F-measure. The horizontal axis represents the number of proposed terms *k* (*k* = 10, 20, …, 500).

The evaluation was performed using the gold standard and the evaluation measures described previously. The evaluation results provided for our method were compared to those achieved by a baseline method. We used TerMine [49], a freely available service from the academic domain based on C-value [24], as the baseline method. The values of all evaluation measures achieved on top *k* (*k* = 10, 20, …, 500) proposed terms are plotted for both methods in Figure 4. Tables 10, 11, 12, 13, 14 illustrate the ATR results by providing top 10 terms as ranked by the two methods. Here we provide a more detailed analysis of the results achieved.

**Table 10:** A comparison to the baseline on data set 1

**Rank**	**FlexiTerm**	**TerMine**
1	transcription factor	t cell
	transcription factors	
	transcriptional factors	
2	nf-kappa b	transcription factor
3	gene expression	nf-kappa b
	expression of genes	
4	transcriptional activity	gene expression
	activator of transcription	
	transcriptional activation	
	activating transcription	
	activators of transcription	
	transcription activation	
	transcriptional activator	
5	nf-kappab activation	cell line
	nf-kappab activity	
6	human t cells	t lymphocyte
	human cells	
7	cell lines	human monocyte
	cell line	
8	human monocytes	dna binding
9	activation of nf-kappa b	tyrosine phosphorylation
	nf-kappa b activation	
	nf-kappa b activity	
10	protein kinase	b cell

Top 10 ranked terms by the two methods.

**Table 11:** A comparison to the baseline on data set 2

**Rank**	**FlexiTerm**	**TerMine**
1	chronic obstructive pulmonary disease	chronic obstructive pulmonary disease
2	patients with copd	obstructive pulmonary disease
	copd patients	
3	pulmonary disease	pulmonary disease
4	acute exacerbation	copd patient
	acute exacerbations	
5	copd exacerbation	acute exacerbation
	copd exacerbations	
	exacerbations of copd	
	exacerbation of copd	
6	patients with chronic obstructive pulmonary disease	severe copd
	patients with chronic obstructive pulmonary diseases	
7	lung function	copd exacerbation
8	exacerbations of chronic obstructive pulmonary disease	lung function
	chronic obstructive pulmonary disease exacerbations	
	exacerbation of chronic obstructive pulmonary disease	
9	quality of life	airway inflammation
10	airway inflammation	exercise capacity

Top 10 ranked terms by the two methods.

**Table 12:** A comparison to the baseline on data set 3

**Rank**	**FlexiTerm**	**TerMine**
1	pulmonary rehab	pulmonary rehab
	pulmanory rehab	
2	breathe easy	breathe easy
3	vitamin d	vitamin d
4	lung transplantation	lung function
	lung transplant	
	lung transplants	
	lung transplantations	
5	breathe easy groups	severe copd
	breath easy groups	
	breathe easy group	
6	chest infection	blood pressure
	chest infections	
7	quality of life	lung disease
8	blood pressure	lung transplant
9	lung function	chest infection
10	rehab room	rehab room

Top 10 ranked terms by the two methods.

**Table 13:** A comparison to the baseline on data set 4

**Rank**	**FlexiTerm**	**TerMine**
1	hospital course	hospital course
	course of hospitalization	
2	chest pain	present illness
3	shortness of breath	chest pain
4	coronary artery	coronary artery
	coronary arteries	
5	present illness	blood pressure
6	blood pressure	ejection fraction
	blood pressures	
7	coronary artery disease	coronary artery disease
8	congestive heart failure	myocardial infarction
9	myocardial infarction	congestive heart failure
10	ejection fraction	cardiac catheterization

Top 10 ranked terms by the two methods.

**Table 14:** A comparison to the baseline on data set 5

**Rank**	**FlexiTerm**	**TerMine**
1	mri knee	collateral ligament
2	collateral ligaments	medial meniscus
3	medial meniscus	lateral meniscus
	medial mensicus	
4	lateral meniscus	hyaline cartilage
5	hyaline cartilage	posterior horn
6	posterior horn	femoral condyle
7	joint effusion	joint effusion
8	mri rt knee	mri lt knee
	mri knee rt	
9	mri lt knee	lateral femoral condyle
	mri knee lt	
10	lateral femoral condyle	medial femoral condyle

Top 10 ranked terms by the two methods.

Our method underperformed on all three evaluation measures only on data set 1, a subset of the GENIA corpus [38]. The precision of our method was worse on the literature data in both domains, i.e. biology (data set 1) and medicine (data set 2). We hypothesise that the better performance of the baseline in terms of precision may stem from the highly regular nature of scientific language in terms of grammatical correctness, e.g. fewer syntactic and typographic errors compared to patient blogs (data set 3) and medical notes (data sets 4 and 5), where the flexibility of our approach in neutralising such errors and other sources of term variation may not be necessarily beneficial. The precision achieved on the remaining data sets does not contradict this hypothesis.

An alternative explanation for better precision of the baseline method is potentially better term candidate extraction prior to termhood calculation since TerMine uses GENIA tagger, which is specifically tuned for biomedical text such as PubMed abstracts [50]. On the other hand, we used Stanford log-linear POS tagger [30,31] using a left-three-words tagging model of general English. This may pose limitation on the performance in the biomedical domain, but also makes the FlexiTerm method more readily portable between domains.

The third reason contributing to poorer precision is the way in which prepositions were annotated in the gold standard and the fact that the baseline method does not include prepositional phrases as part of term candidates. Our method does recognise prepositional phrases as term components, which in effect will tend to favour longer phrases such as *exacerbation of chronic obstructive pulmonary disease* recognised by our method, but not the baseline (see Table 11). Due to the problems with complexity and subjectivity associated with the annotation of compound terms (i.e. the ones which contain nested terms) as explained in the previous subsection, prepositions are likely not to be consistently annotated. In the given example this means that if one annotator failed to annotated the whole phrase and instead annotated *exacerbation* and *chronic obstructive pulmonary disease* as separate terms, the preposition *of* would be counted as a false positive in token-level evaluation. Therefore, prepositions that are syntactic constituents of terms partly account for the drop in precision. However, prepositions do need to be considered during term recognition and this in fact may boost the performance in terms of both precision and recall. We illustrate this point by the following examples. Data sets 2 and 3 are in the same domain (COPD), but written from different perspectives and by different types of authors. As they share the same domain, they naturally share some of the terminology used. Tables 11 and 12 show that the phrase *quality of life* is ranked highly by our method in both data sets. We checked the terminological status of the hypothesised term by looking it up in the UMLS where it is indeed defined as "*A generic concept reflecting concern with the modification and enhancement of life attributes, e.g., physical, political, moral and social environment; the overall condition of a human life.*" Nonetheless, the inspection of the complete results proved that the baseline method does not recognise it at all. The results on data set 4 (see Table 13) provide a similar example, *shortness of breath*, listed as a synonym of *dyspnea* in the UMLS, which was ranked third by our method, but again not recognised at all by the baseline. Failure to include prepositions therefore may completely overlook extremely important concepts in a domain. In less extreme cases, it may skew the term recognition results with less severe but still significant effects. For example, the difference in ranking of *copd exacerbation* in data set 2 may not seem significant. It was ranked seventh by the baseline method and slightly higher at five by our method due to the fact that the information obtained for two variants *copd exacerbation* and *exacerbation of copd* was aggregated. The difference in ranking of the same term in data set 3, where it is used less often, becomes more prominent (16 in our method compared to 47 in the baseline method), thus signifying the importance of aggregation for sparse data.

The importance of aggregation is nicely illustrated with the increase of precision in data set 5 (see Table 14), which exhibits high degree of derivational and orthographic variation often as a result of typographical errors. For example, the third ranked term *medial meniscus* also includes its misspelled variant *medial mensicus*, which otherwise would not be recognised in isolation due to its low frequency. The 11th ranked term includes two orthographic variants *postero-lateral corner* and *posterolateral corner* in our results, while the baseline method ranks them separately at 18 and 55 respectively. Another interesting example is the 14th ranked term, which includes three variants *infrapatellar fat pad*, *infra-patella fat pad* and *infra-patellar fat pad*, the first one ranked 20th by the baseline method and the remaining two ranked as low as 281. The results on this data set demonstrate how flexible aggregation of term variants with the same or related meaning can significantly improve the precision of ATR (see Figure 4).

In general, with the exception of the literature data sets, the precision of our method is either comparable (an improvement rate of 0.71 percentage points on data set 3) or better (an improvement rate of 2.02 and 3.29 percentage points on data sets 4 and 5 respectively) than that of baseline. The natural drop in precision as the recall increases also seems to be less steep on all five data sets. Interestingly, the precision of both methods is rising on data set 4 and very soon stabilises to almost constant level. On another type of clinical text data (data set 5) where the recall values were nearly identical, the aggregation of term variants and their frequencies significantly boosts the precision as the recall increases.

A similar effect can be observed in boosting the recall, which is either comparable (a drop by 0.96 percentage points on data set 5) or better than the baseline (an improvement rate of 3.77, 3.96 and 2.43 percentage points on data sets 2–4 respectively). The boost in recall is most obvious on terminologically sparse data set 3. When precision and recall are combined, the F-measure is better than that of the baseline with the exception of data set 1. It is significantly better on data sets 3 and 4 (an improvement rate of 2.73 and 2.77 percentage points respectively) where both precision and recall were improved.

In conclusion, both methods perform comparably well on literature and clinical notes. However, based on the results achieved on data set 3, it appears that the flexibility incorporated into the FlexiTerm method makes it more robust for less formal types of text data where the terminology is sparse and not necessarily used in the standard way. The underperformance on data set 1 in comparison to performance on other data sets does show that the results on this corpus cannot be generalised for other biomedical domains and language types as suggested in [37].

### Computational efficiency

Computational efficiency of FlexiTerm is a function of three variables: the size of the dataset, the number of term candidates and the number of unique stemmed tokens that are part of term candidates. The size of the dataset will be reflected in the time required to linguistically pre-process all documents, including POS tagging and stemming. Additional time will be spent on term recognition including the selection of term candidates based on a set of regular expressions and their normalisation based on token similarity. Similarity calculation is the most computationally intensive operation and its complexity is quadratic to the number of unique stemmed tokens extracted from term candidates. According to Zipf's law, which states that a few words occur very often while others occur rarely, the number of unique tokens is not expected to rise proportionally with the corpus size. Therefore, the similarity calculation should not affect the scalability of the overall approach. Table 15 provides execution times recorded on five datasets used in evaluation.

**Table 15:** Computational performance

**Data set**	**Linguistic pre-processing**	**Term recognition**
1	14 sec	101 sec
2	13 sec	96 sec
3	10 sec	59 sec
4	26 sec	290 sec
5	12 sec	32 sec

Completion times across five datasets.

## Conclusions

In this paper, we described a new ATR approach and demonstrated that its performance is comparable to that of the baseline method. Substantial improvement over the baseline has been noticed on sparse and non-standardised text data due to the flexibility in the way in which termhood is calculated. While the syntactic structure of terms is an important factor in distinguishing between terms and non-terms, the results show that it need not be part of termhood calculation. Therefore, we suggest that the analysis of syntactic structure should be confined to linguistic filters used to select term candidates, after which they should be treated using a bag-of-word approach.

We also suggest grouping semantically related term candidates to further improve the termhood calculation for sparse terms. Such grouping can be achieved using phonetic and lexical similarity as a proxy for semantic similarity. Further improvement of semantic grouping can be achieved by using other methods to measure semantic relatedness between words. Latent semantic analysis, which statistically analyses contextual information over a large corpus in order to link related words [51], is an obvious choice and incorporating it into the FlexiTerm framework will be the subject of future work. To further improve the results of terminologically processing the data retrieved from the Web, we will conduct experiments with the Google distance [52], a semantic similarity measure calculated as a function of hits returned by the Google search engine for the given words, where words with similar meaning tend to appear close in this measure.

The improved performance of term recognition on data obtained from the Web and social media in particular may facilitate consumer health informatics research [53] by efficiently extracting consumer health vocabulary [54], thus effectively bridging the consumer-professional gap in communication. The extracted terminology can support traditional qualitative research techniques such as content analysis (e.g. [55,56]) by highlighting the most important concepts mentioned. More importantly, it can support large-scale processing with text mining. For example, ATR in combination with sentiment analysis can quickly reveal major concerns faced by specific patient populations, thus providing essential information for health policy makers beyond that obtained with the traditional survey techniques.

### Availability and requirements

**Project name:** FlexiTerm

**Project home page:**http://www.cs.cf.ac.uk/flexiterm

**Operating system(s):** Platform independent

**Programming language:** Java

**Other requirements:** None

**License:** FreeBSD

**Any restrictions to use by non-academics:** None

## Competing interests

To the best knowledge of the authors, there are no competing interests.

## Authors’ contributions

IS conceived the overall study, designed and implemented the application and drafted the manuscript. MG contributed to the implementation, collected the data and coordinated the evaluation. AP was consulted throughout the project on all development issues. NF and GE lent their medical expertise to interpret the results. All authors read and approved the final manuscript.
